# Associations among Employment Status, Health Behaviors, and Mental Health in a Representative Sample of South Koreans

**DOI:** 10.3390/ijerph17072456

**Published:** 2020-04-03

**Authors:** Se Jin Park, Soo Yeon Kim, Eun-Sun Lee, Subin Park

**Affiliations:** Department of Research Planning, Mental Health Research Institute, National Center for Mental Health, Seoul 04933, Korea; satang95@korea.kr (S.J.P.); oss1793@daum.net (S.Y.K.); chachagrace918@gmail.com (E.-S.L.)

**Keywords:** employment status, health behavior, general health, mental health

## Abstract

The purpose of the present study was to compare the health behaviors, general health, and mental health of South Korean employees according to their employment status, and to examine how these associations vary across genders using the latest Korean National Examination Health and Nutrition Survey data. Logistic regression analyses were performed using employment status—permanent job, temporary job, and unemployed—as predictor variables and health-related variables as the outcome variables. Results indicated that temporary workers and the unemployed have higher odds of poor mental health regardless of gender. On the other hand, only male permanent workers were found to have a higher risk of problematic drinking compared to precarious workers and the unemployed. Meanwhile, only women showed a higher risk of current smoking in the temporary job and unemployed groups compared with permanent employees. Regarding general health, women, not men, in the temporary job group reported poorer general health (i.e., low health-related quality of life and higher self-perceived poor health) than those in other groups. These findings suggest that the development and implementation of intervention services, as well as organizational actions, need to consider differential impacts of unfavorable employment status on health issues according to gender.

## 1. Introduction

Globalization, deindustrialization, and economic crises have led to socioeconomic changes in modern society, which have also brought about labor market flexibility [1]. Labor market flexibility has transformed the structure of the labor market by diversifying employment relationship to include alternative employment instead of only standard employment. Alternative employment refers to employment that is flexible, temporary, irregular, non-standard, or precarious [2]. Benach and colleagues [3] defined precarious employment as “a multidimensional construct encompassing dimensions such as employment insecurity, individualized bargaining relations between workers and employers, low wages and economic deprivation, limited workplace rights and social protection, and powerlessness to exercise work place rights.” Precarious employment possesses the following characteristics: lack of “standard” employment benefits, short or uncertain duration, lack of protective regulation, and ambiguous or unprotected legal status [4].

According to Organization for Economic Cooperation and Development (OECD) statistics [5], the present employment rate in South Korea (hereinafter Korea) is 66.9%, similar to the average employment rates of other OECD countries (68.7%). However, the youth (aged 15–24 years) employment rate in Korea is 26.3% at present, which is much lower than the average youth employment rate (42.3%) of other OECD countries [6,7]. Furthermore, the temporary employment rate in South Korea is 16.8%, which is higher than the average temporary employment rate of OECD countries (11.7%) [6,7]. This statistics indicate that temporary employment and youth unemployment are serious social problems in Korea [8].

The participation of women in economic activities has steadily increased due to socioeconomic changes and the increasing awareness regarding social participation [9]. However, the higher rate of women’s employment has not improved the status of women in society, and gender discrimination continues to be rampant [9]. Due to the power structure in society that favors men (i.e., patriarchy), as well as the gender division of the labor market, women are likely to be marginalized, unstable, and low-wage or non-standard job workers with inconstant working forms such as temporary employment, regular part-time work, and on-call/day labor [10]. In Korea, for example, 41.5% of the female work force was involved in non-standard work arrangements (i.e., temporary, regular part-time, and on-call/day labor) in 2018, compared to only 26.3% of the male work force [11].

Precarious workers suffer from lack of social protection, including detrimental physical and psychosocial working conditions, weaker occupational health and safety measures, and under-protection from social risks such as unemployment, incapacity, and retirement. Consequently, such social precariousness may result in material deprivation along with its associated health consequences [12]. For example, compared to permanent employees, temporary employees were found to have significantly worse physical health, such as drinking- and smoking-related mortality and musculoskeletal disorders [3], as well as mental health problems such as depression, psychological fatigue, and sleep disorder [3,13]. In addition, it has been reported that part-time employment and unemployment are strongly associated with lower life satisfaction, as compared to full-time employment [14]. Moreover, a number of gender-issue researchers suggest that precarious employment, in combination with gender-related historical factors such as patriarchy, may damage women’s health more than that of men [10]. Women are more employed in non-standard jobs of a caring nature—such as home-care workers or nurse assistants—in which they are often expected to work overtime and endure abuse from clients because of the gendered nature of the labor (e.g., giving, selfless, suffering, nurturing) [10]. Additionally, irrespective of how high their occupational status is, women rarely have the authority to oblige men to undertake an equal share of domestic labor and childcare [15].

Considering the growing trend of precarious employment in Korea [8] as well as the public health consequences of precarious and non-standard employment [12], more research that reflects such domestic situations is needed in this area [16,17]. However, in Korea, there is insufficient research on these issues. Therefore, the present study aimed to compare the health behaviors as well as the general and mental health of South Korean employees based on their employment status using data from the latest Korean National Examination Health and Nutrition Survey. Furthermore, we examined how these associations between precarious employment and health-related variables may differ between men and women.

## 2. Methods

### 2.1. Study Sample

This study was performed using data obtained from the Korean National Health and Nutrition Examination Survey (KNHANES VI and VII, 2013–2017). This cross-sectional survey was conducted on a nationally representative sample of Korean civilians by the Korean Centers for Disease Control and Prevention (KCDC). The survey employed a complex, stratified, and multistage probability sampling model, with sampling units comprising an average of 60 households. Altogether, 20 households per sampling unit were sampled through intra-stratification based on geographical area, age, and sex [18]. The KNHANES comprised a health interview, health examination, and a nutrition survey. The health interview was performed by a trained interviewer using a self-administered and structured questionnaire to obtain information regarding participants’ socio-demographic characteristics, health behaviors, and (mental) health status. The Institutional Review Board of KCDC approved the survey protocol.

**Figure 1:**
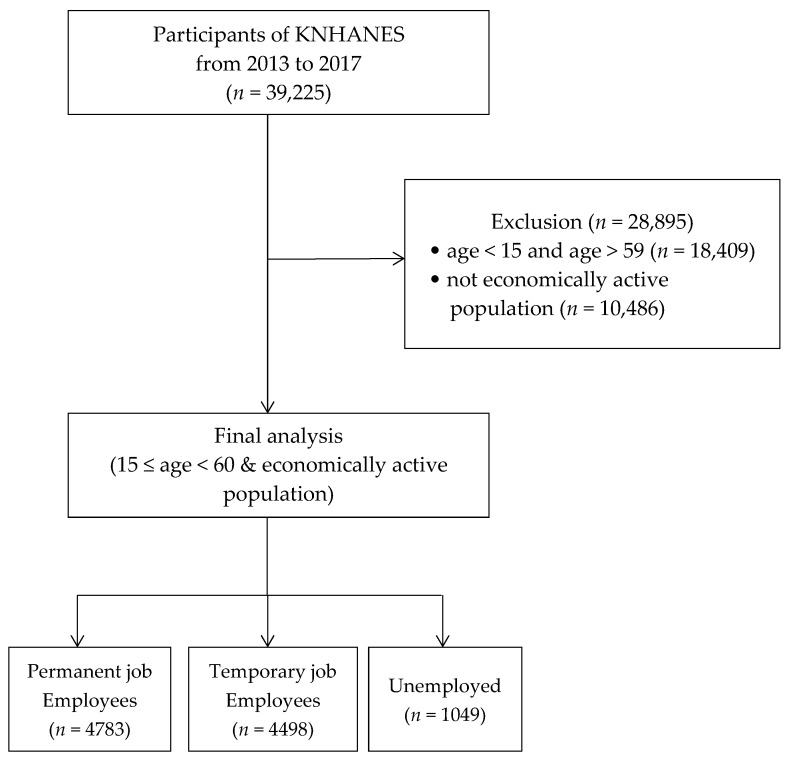
Flow diagram of selection of study participants. Abbreviations: KNHANES, Korean National Health and Nutrition Examination Survey.

Of the 39,225 participants in the KNHANES from 2013 to 2017, we included 10,330 economically active participants in the age range of 15–59 years, which included both employed participants and unemployed ones seeking jobs. We created three categories based on employment status: 4783 participants with permanent jobs, 4498 with temporary jobs, and 1049 unemployed (Figure 1).

### 2.2. Measures

Each participant’s employment status was categorized into permanent job, temporary job, and unemployed based on the questionnaire responses. Permanent and temporary job included only wage workers, excluding those who were self-employed, employers, and unpaid family workers. Unemployed was defined as people who do not have jobs, but are seeking employment at present.

Mental health variables included depression (depressed mood, depression symptoms, and depression diagnosis), suicidality (suicide ideation, planning, and attempt), and daily perceived stress. The information on mental health was based on participants’ self-reports. Depressed mood was defined as an individual experiencing sadness or despair that disrupted their daily lives continuously for over 2 weeks in the past year (i.e., responding “yes” to the question, “Have you felt sadness or despair affecting your daily life for more than 2 weeks over the past year?”). Depression symptoms were assessed using the Patient Health Questionnaire (PHQ-9), which comprises nine items rated from 0 (not at all) to 3 (experiencing symptoms nearly every day); the scores for each item are summed to produce the total depression severity score (range: 0–27). The Korean version of the PHQ-9 demonstrates high internal consistency (Cronbach’s α = 0.86), and the optimal cutoff total score for the presence of depression is 5 [19]. A PHQ-9 score ≥10 was defined as indicating depression symptoms. Depression diagnosis was defined as having been clinically diagnosed with depression by a physician at some point during their lives (i.e., responding “yes” to the question “Have you ever been diagnosed with depression by a physician?”). Suicidal ideation, planning, and attempt were assessed by the questions, “Have you ever seriously considered suicide during the past year?”, “Have you ever made a specific plan to commit suicide during the past year?”, and “Have you ever actually attempt suicide during the past year?”, respectively, with responses of “yes” and “no”. Daily perceived stress was measured by the question, “How do you usually feel stress in your daily life?” with responses provided on a 4-point Likert scale (very high, high, low, or little). Participants who responded with “very high” or “high” were defined as having high daily perceived stress.

General health included items on health-related quality of life (HRQoL) and self-perceived health. HRQoL was measured using the EQ-5D (EuroQol-5D) questionnaire, which comprises items on five dimensions—mobility, self-care, usual activities, pain/discomfort, and anxiety/depression. There were three responses (1 = no problem, 2 = moderate problem, and 3 = severe problem) for each of the five questions. The EQ-5D index scores range from −0.171 on a scale in which 0 indicates death and 1.0 indicates perfect health (a negative score demonstrates health status worse than death). The Korean version of the EQ-5D has been established, and its reliability and validity have been proven [20]. Self-perceived health status was assessed by the question, “Generally, how is your subjective physical health status?” with responses provided on a 5-point Likert scale (very poor, poor, fair, good, or very good). Participants who responded with “very poor” or “poor” were defined as those with poor self-perceived health.

Health behaviors included smoking status (currently a smoker, yes or no), alcohol consumption (high-risk consumption, yes or no), sleep time, and mental health service use. For sleeping duration, data on the average time (unit: minute) spent sleeping per day on weekdays was collected, and participants who did not sleep for 6–8 hours per day were defined as those with an unhealthy sleep time. Mental health service use was assessed by the question, “Have you visited any healthcare institutions, or have you received consultation through the Internet, telephone, etc. due to your mental health problems during the past year?”, with the responses “yes” or “no”.

Socio-demographic factors included education (< college and ≥ college), monthly household income (<quartile 1, quartile 1 to quartile 2, quartile 2 to quartile 3, and ≥quartile 3), sex, age group (15–19, 20–29, 30–39, 40–49, 50–59), and marital status (married, divorced/widowed/separated, and never married).

### 2.3. Statistical Analysis

Weighted values were applied using the survey-related procedure in the SAS software version 9.4 (SAS Institute Inc., Cary, NC, USA), considering that the KNHANES features complex sampling. Socio-demographic characteristics, health behaviors, and health outcomes were compared by participants’ employment status (permanent vs. temporary job, permanent job vs. unemployed, and temporary job vs. unemployed) using the chi-square test and analysis of variance (ANOVA). To determine the relationship between employment status and health (mental and general health), multiple logistic regression (outcome: binomial variable) and multiple regression (outcome: continuous variable, i.e., HRQoL) were used to calculate the adjusted odds ratio (AOR), 95% confidence intervals (95% CIs), and coefficient and standard errors (SE), respectively, after adjusting for socio-demographic characteristics. The cut-off for significance was set at *p* < 0.05 for all analyses.

## 3. Results

Table 1 presents the results of the descriptive analysis of the study sample according to employment status. The sample comprised 5105 male and 5225 female participants, with a mean age of 37.3 (standard deviation (SD) = 2.5 years). 46.0% of all participants had permanent jobs, 43.3% were temporary job workers, and 10.7% were unemployed (job seekers). There were significant group differences in the socio-demographic characteristics of participants (i.e., age, age group, gender, education, monthly income, and marital status) according to employment status.

Table 2 presents descriptive information regarding health variables according to employment status. In the comparison between the permanent and temporary job groups, significant differences were found in all variables, except in the variable of high daily perceived stress. In the comparison between permanent and unemployed groups, all variables except self-perceived health, high daily perceived stress, and suicide attempt factors demonstrated significant differences. In the comparison between temporary job and unemployed groups, high-risk alcohol consumption, sleeping duration per day, unhealthy sleeping duration, and depressed mood factors showed significant differences.

Table 3 provides the results of the binary logistic regression analyses, after adjusting for the participants’ ages, education levels, household incomes, and marital status. In health behaviors, the permanent job group demonstrated more high risky alcohol consumption than the unemployed group, and was found to be receiving fewer mental health services than the other two groups. Furthermore, the temporary job and unemployed groups reported unhealthy sleeping duration more than the permanent job group. Men with permanent jobs, compared to those from the other two groups, exhibited significantly higher odds of high-risk alcohol consumption. Women in the temporary job and unemployment groups showed significantly higher odds of current smoking than those in the permanent job group.

For general health, the temporary job and unemployed groups reported lower health-related quality of life than the permanent job group. When analyzed separately for gender, there was a significant association found between having a temporary job or being unemployed and lower health-related quality of life in women, but not in men. Women in the unemployed group also reported significantly lower self-perceived health than those in the permanent job group.

For mental health, men and women in both the temporary job and unemployed group reported significantly higher depression (e.g., depressed mood, depression symptoms, and being diagnosed with depression), and suicidality (e.g., suicidal ideation or planning) than those in the permanent job group. There were no significant differences found in general and mental health between the temporary job and unemployed groups both in men and women.

## 4. Discussion

The purpose of this study was to compare the health behaviors and mental health of employees according to their employment status using a nationally representative sample of Koreans. The study findings suggest that the mental health (e.g., depression, suicidality, and sleeping duration) as well as general health of the temporary job and unemployed groups are worse than those of the permanent job group; in addition, the associations between health behaviors, general health, and employment status are different for both genders.

In this study, 10.7% of the participants were unemployed, and 48.4% of those in the unemployed group were younger than 20. Meanwhile, 43.3% of the participants had temporary jobs, and 34.8% of those in the temporary job group were younger than 20. These findings indicate that precarious employment among the youth should be considered a serious social problem in Korea. It is noteworthy that young adults are particularly vulnerable in the labor market as they lack work experience [21], and consequently face more insecure and unsuitable working conditions [11].

Regarding health behaviors, in men, those in the permanent and temporary job groups were found to exhibit a higher risk of problematic drinking than those in the unemployed group, while women did not significantly differ with regard to high-risk drinking according to employment status. These gender differences in drinking among employees could reflect the unique Korean drinking culture. For Korean males, drinking alcohol is a primary coping method used to increase sociability, strengthen interpersonal relationships, and reduce tension; thus, it is common for employees to drink alcohol with their coworkers or friends after work [22]. Moreover, employers’ encouragement of social gatherings that involve alcohol consumption among employees, as well as the leniency when it comes to offenses committed by workers who are dining and drinking together could contribute to the excessive alcohol consumption [23]. Furthermore, the risk for excessive alcohol consumption tends to increase as male workers get older and promoted, leading to more social gatherings in the workplace [24]. Thus, companies and mental health professionals need to develop and implement prevention and intervention programs that track the risk for problematic alcohol consumption among male employees, help them maintain sound drinking habits and acquire healthy stress-relieving strategies by providing professional guidance.

On the other hand, women in the temporary job and unemployed groups were found to demonstrate a higher risk of current smoking than those in the permanent job group, while male participants did not show significant difference in the risk of smoking. This may point to the possibility that women’s health behaviors are more subject to their current employment status than those in men. This is in line with the result of a previous study that indicated a higher effect of demographics on smoking in women [25]. Whereas male smokers tend to not be concentrated in specific social classes, women with lower education levels, precarious employment, and divorced, widowed, or separated women exhibit a higher rate of smoking than those in the other groups [25]. This result implies that intensive smoking prevention and anti-smoking policies are necessary for women with a vulnerable socioeconomic status.

Regarding mental health, the temporary job and unemployed groups reported worse mental health (i.e., depression and suicidal ideation or planning) than the permanent job group for both men and women. Nevertheless, mental health services use rates were very low across all employment statuses, ranging from 1.6% to 4.6%, which are far lower than the prevalence rate for 12-month help-seeking (8.3%) found in the general population in Western countries [26]. This may be due to the negative opinions about professional help-seeking (e.g., stigma) in Asian culture [27]. Furthermore, men with a temporary employment or unemployed status did not differ from permanent employees in the degree of mental health services use, whereas, in women, temporary employees or the unemployed showed a significantly higher usage of mental health services. The gender difference in mental health services use could be derived from findings that women exhibit a positive attitude toward professional mental health services as compared to men [28,29,30]. Moreover, it has been reported that Korean men tend to avoid personal psychological problems through drinking behaviors [31]. This result implies the need for the development and implementation of additional guidance that helps to lower the psychological barriers to professional help targeting male employees.

In addition, the female, but not the male, temporary job group reported poor general health (i.e., low HRQoL and self-perceived poor health). This implies that women in precarious employment situations are prone to experience more substantial physical discomfort and limitations of usual activities, which add to the burden of poor mental health due to a temporary job status and contribute to a lower life satisfaction. In general, women are pressured to take on the responsibility of family life and parenting in addition to working. In this regard, women’s participation in precarious employment is often involuntary, with no discretion, due to family constraints (e.g., lack of alternative childcare options), which has a significantly negative impact on subjective well-being [14]. Another reason may be that there are differences between men and women in terms of the contract and working conditions, even in the same working environment [9,10]. Women who perceived unsatisfactory working conditions reported lower life satisfaction and higher physical distress and anxiety than women with permanent jobs and unemployed women; however, the men did not [32]. Our findings imply that the effect of employment status on physical and mental health may be different by gender. Therefore, intervention programs for women should be conducted in tandem with the present societal context (e.g., characterized by patriarchy and employment discrimination). For instance, providing on/off-line occupational education or job skills training for women who inevitably have to work part-time could off-set the psychological costs of having to choose precarious employment.

The findings of the present study have several implications. First, this study performed a comparison of health behaviors and general and mental health according to employment status using the Korean National Examination Health and Nutrition Survey data with national representation. Second, we found that mental health varies based on employment status. Specifically, the aspects of mental health vary between men and women, even if they have the same employment status. This suggests that traditional recognition of social roles (e.g., breadwinners, and patriarchy) should be considered while conducting psychological interventions for women. We believe that our findings will help experts in related fields and employment support programs.

Despite the notable findings, this study has several limitations. First, this study only considered age, gender, income, education levels, and marital status as various demographic socioeconomic factors. Of note, the degree of voluntariness in or preference for choosing contingent work as well as occupational type of a precarious position (e.g., professional and knowledge workers) may also influence precarious employees’ health status [33]. Therefore, further studies should consider other socioeconomic factors (e.g., region, social status), as well as other important aspects of precarious employment (e.g., whether an individual chose the position voluntarily). Second, the data used in this study were cross-sectional data, which were collected at a point in time; therefore, the causal relationship between the studied variables could not be revealed. Furthermore, given that indicators of health behaviors, general health, and mental health are correlated, future studies need to examine the effect of employment status on those variables after adjusting for the effects they have on each other. Third, we could not identify the differences in mental health due to changes in employment status over time. For example, the difference in mental health between someone who recently became a permanent employee and another who had already been a permanent employee for a long time has not been clarified. Finally, because the Korean National Examination Health and Nutrition Survey data was conducted using self-report questionnaires, there are information biases regarding socioeconomic status or health behavior responses such as occupation, education level, and income, which may cause differential misclassification. Longitudinal studies of longer duration and with more various socioeconomic factors taken into consideration should be performed to verify our findings.

## 5. Conclusions

This study compared the multiple aspects of health (health behaviors as well as general and mental health) of participants according to their employment status using a nationally representative sample of Koreans, examining how these associations differ according to gender. Altogether, 10.7% of the participants were unemployed, and 48.4% of those in the unemployed group were younger than 20. Meanwhile, 43.3% of the participants had temporary jobs, and 34.8% of those in the temporary job group were younger than 20. The mental health (e.g., depression, suicidality, and sleeping duration) as well as general health of those in the temporary job and unemployed groups was worse than that of those in the permanent job group; in particular, men in temporary job positions did not use mental health services even though they reported poor mental health. In addition, women in the temporary job and unemployed group reported poorer general health than those in the permanent employment group. The findings are meaningful in that they suggest the importance of considering gender differences, as well as the present societal context (e.g., patriarchy and employment discrimination), on the impact of precarious employment on health associations.

## Figures and Tables

**Table 1:** Descriptive analysis of the study sample according to employment status.

Socio-Demographic Variables	Permanent Job *N* = 4783 (46.0%)		Temporary Job *N* = 4498 (43.3%)		Unemployed *N* = 1049 (10.7%)		Permanent Job vs. Temporary Job	Permanent Job vs. Unemployed	Temporary job vs. Unemployed
	*n*	%	*n*	%	*n*	%			
**Age, mean**	39.7	0.2	37.4	0.2	34.7	0.4	<0.0001	<0.0001	<0.0001
**Age group, year**									
15–19	13	0.3	284	6.9	63	5.2	<0.0001	<0.0001	<0.0001
20–29	651	16.5	964	27.9	359	43.2			
30–39	1613	34.0	862	18.9	192	17.1			
40–49	1548	31.1	1110	23.1	196	15.6			
50–59	958	18.1	1278	23.2	239	18.9			
**Gender**									
Men	2994	69.6	1672	45.5	439	50.0	<0.0001	<0.0001	<0.0257
Women	1789	30.4	2826	54.5	610	50.0			
**Education**									
High school degree or less	1584	33.0	2923	64.7	542	48.5	<0.0001	<0.0001	<0.0001
College degree or more	3197	67.0	1574	35.3	506	51.5			
**Monthly household income**									
<Q1	82	1.6	432	9.9	189	19.0	<0.0001	<0.0001	<0.0001
Q1 to <Q2	689	14.3	1335	29.3	330	30.2			
Q2 to <Q3	1635	35.5	1533	34.5	304	29.4			
≥Q3	2368	48.7	1190	26.3	216	21.4			
**Marital status**									
Married	3632	73.4	2544	50.9	407	32.8	<0.0001	<0.0001	<0.0001
Divorced, Widowed, or Separated	167	3.0	403	7.3	72	5.2			
Never married	980	23.6	1544	41.8	569	62.0			

**Table 2:** Health status of participants according to employment status.

Health-Related Variables	Permanent Job *N* = 4783 (46.0%)		Temporary Job *N* = 4498 (43.3%)		Unemployed *N* = 1049 (10.7%)		Permanent Job vs. Temporary Job	Permanent Job vs. Unemployed	Temporary job vs. Unemployed
	*n*	%	*n*	%	*n*	%			
**Health behaviors**									
Current smoking (yes)	1296	30.3	939	25.3	219	23.8	<0.0001	0.0005	0.3974
High risk alcohol consumption (yes)	766	17.6	598	14.2	111	10.5	0.0003	<0.0001	0.0044
Sleeping duration (hours) per day									
<4 hours	8	0.3	20	1.1	3	0.6	<0.0001	<0.0001	0.0023
4–6 hours	280	13.3	256	12.8	58	11.6			
6–8 hours	1481	67.5	1094	57.5	233	49.3			
≥8 hours	438	19.0	527	28.6	168	38.5			
Unhealthy sleeping duration	726	32.5	803	42.5	229	50.7	<0.0001	<0.0001	0.0023
Mental health service use (yes)	53	1.6	100	3.2	32	4.6	<0.0001	<0.0001	0.1195
**General health**									
Health-related quality of life (EQ-5D index), mean (standard error, SE)	0.98	0.01	0.97	0.01	0.97	0.01	<0.0001	<0.0001	0.9965
Self-perceived poor health, mean (SE)	454	9.5	487	10.8	138	13.0	0.0725	0.0014	0.0573
**Mental health**									
High daily perceived stress (yes)	1426	29.8	1382	31.3	304	29.3	0.1715	0.7927	0.2709
Depressed mood (yes)	186	6.0	329	11.5	97	15.7	<0.0001	<0.0001	0.0102
Depression symptoms (PHG-9 ≥10) ^a^ (yes)	52	2.5	96	5.7	32	7.4	<0.0001	<0.0001	0.2801
Ever been diagnosed with depression (yes)	64	1.2	187	4.5	41	3.4	<0.0001	<0.0001	0.1188
Suicidal ideation or planning (yes)	60	1.2	151	4.2	38	4.6	<0.0001	<0.0001	0.684
Suicide attempt (yes)	8	0.1	19	0.5	4	0.4	0.0015	0.0755	0.8024

Abbreviations: EQ-5D, EuroQol-5D; ^a^ PHG-9, Patient Health Questionnaire.

**Table 3:** Association between employment status and health after adjusting for the explanatory variables.

Health-Related Variables	Total			Men			Women		
	Temporary Job vs. Permanent Job (Ref)	Unemployed vs. Permanent Job (Ref)	Unemployed vs. Temporary Job (Ref)	Temporary Job vs. Permanent Job (Ref)	Unemployed vs. Permanent Job (Ref)	Unemployed vs. Temporary Job (Ref)	Temporary Job vs. Permanent Job (Ref)	Unemployed vs. Permanent Job (Ref)	Unemployed vs. Temporary Job (Ref)
	AOR (95% CI)	AOR (95% CI)	AOR (95% CI)	AOR (95% CI)	AOR (95% CI)	AOR (95% CI)	AOR (95% CI)	AOR (95% CI)	AOR (95% CI)
**Health behavior**									
Current smoking	1.05 (0.92–1.21)	0.93 (0.73–1.17)	0.88 (0.70–1.11)	1.01 (0.86–1.18)	0.91 (0.70–1.17)	0.90 (0.70–1.16)	1.81 (1.30–2.51)	1.63 (1.10–2.66)	0.90 (0.59–1.38)
High risky alcohol consumption	0.99 (0.84–1.16)	0.70 (0.53–0.91)	0.70 (0.54–0.91)	1.02 (0.84–1.24)	0.67 (0.48–0.93)	0.66 (0.47–0.92)	1.22 (0.92–1.61)	1.04 (0.68–1.61)	0.86 (0.58–1.27)
Unhealthy sleeping duration (yes)	1.36 (1.15–1.60)	1.80 (1.42–2.28)	1.33 (1.07–1.66)	1.34 (1.04–1.72)	1.95 (1.32–2.87)	1.46 (0.99–2.14)	1.39 (1.10–1.74)	1.71 (1.22–2.39)	1.23 (0.91–1.66)
Mental health service use	1.63 (1.09–2.44)	2.43 (1.44–4.11)	1.49 (0.92–2.42)	0.94 (0.45–1.98)	2.19 (0.94–5.10)	2.33 (1.10–4.95)	2.27 (1.36–3.77)	2.50 (1.21–5.16)	1.10 (0.57–2.13)
**General health**									
Health-related quality of life (EQ-5D index), coefficient (SE)	−0.005 ** (0.001)	−0.007 ** (0.002)	−0.002 (0.002)	−0.004 (0.002)	−0.006 (0.003)	−0.003 (0.003)	−0.006 ** (0.002)	−0.006 * (0.003)	−0.001 (0.003)
Self-perceived poor health, coefficient (SE)	1.17 (0.98–1.39)	1.54 (1.21–1.97)	1.32 (1.06–1.65)	1.10 (0.87–1.39)	1.40 (0.98–2.01)	1.28 (0.92–1.89)	1.17 (0.90–1.51)	1.56 (1.09–2.22)	1.34 (0.98–1.83)
**Mental health**									
High daily perceived stress	1.06 (0.95–1.19)	0.90 (0.76–1.07)	0.85 (0.72–1.01)	1.19 (1.01–1.41)	1.00 (0.75–1.32)	0.84 (0.64–1.10)	1.04 (0.90–1.21)	0.93 (0.74–1.17)	0.89 (0.72–1.10)
Depressed mood	1.39 (1.11–1.73)	1.88 (1.37–2.60)	1.36 (1.02–1.81)	1.34 (0.93–1.94)	1.81 (1.07–3.05)	1.35 (0.85–2.14)	1.32 (0.99–1.76)	1.79 (1.18–2.71)	1.35 (0.93–1.97)
Depression symptoms (PHQ-9 ≥10 score)	1.70 (1.10–2.62)	2.07 (1.24–3.46)	1.22 (0.76–1.97)	2.51 (1.25–5.04)	1.71 (0.61–4.78)	0.68 (0.26–1.77)	1.49 (0.89–2.47)	2.46 (1.31–4.62)	1.66 (0.93–1.97)
Ever been diagnosed with depression	2.78 (1.99–3.89)	2.38 (1.52–3.72)	0.85 (0.58–1.27)	2.42 (1.42–4.11)	2.88 (1.37–6.04)	1.19 (0.61–2.32)	2.79 (1.82–4.28)	1.82 (1.00–3.33)	0.65 (0.40–1.06)
Suicidal ideation or planning	2.06 (1.42–3.01)	2.34 (1.36–4.04)	1.14 (0.73–1.77)	2.10 (1.14–3.87)	3.47 (1.55–7.77)	1.65 (0.87–3.14)	1.90 (1.19–3.05)	1.51 (0.74–3.09)	0.80 (0.43–1.47)
Suicide attempt	1.65 (0.66–4.17)	1.57 (0.33–7.34)	0.95 (0.30–3.04)	1.48 (0.39–5.58)	3.57 (0.42–30.04)	2.42 (0.52–11.13)	1.22 (0.37–4.09)	No result	No result

The permanent job group was treated as the reference. Abbreviations: EQ-5D, EuroQol-5D; PHG-9, Patient Health Questionnaire; AOR, adjusted odds ratio; adjusting for age, education level, household income, and marital status; CI, confidence interval, * *p* < 0.05, ** *p* < 0.01.
